# Ruscogenins Improve CD-Like Enteritis by Inhibiting Apoptosis of Intestinal Epithelial Cells and Activating Nrf2/NQO1 Pathway

**DOI:** 10.1155/2022/4877275

**Published:** 2022-03-10

**Authors:** Hexin Wen, Xiaofeng Zhang, Qingqing Li, Ju Huang, Guangyong Liu, Jingyue Zhao, Yiran Liu, Li Shen, Yuyang Li, Kun Yang, Lugen Zuo, Jing Li, Jing Nian, Ping Xiang, Hao Zhao, Liang Yu, Mulin Liu, Zhijun Geng, Xue Song

**Affiliations:** ^1^Department of Gastrointestinal Surgery, First Affiliated Hospital of Bengbu Medical College, Bengbu, Anhui, China; ^2^Anhui Key Laboratory of Tissue Transplantation, Bengbu Medical College, Bengbu, China; ^3^Department of Central Laboratory, First Affiliated Hospital of Bengbu Medical College, Bengbu, Anhui, China; ^4^Department of Clinical Medicine, Bengbu Medical College, Bengbu, China; ^5^Department of Clinical Laboratory, First Affiliated Hospital of Bengbu Medical College, Bengbu, China; ^6^Department of Imaging, Second Affiliated Hospital of Bengbu Medical College, Bengbu, Anhui, China

## Abstract

Interaction of intestinal barrier dysfunction and intestinal inflammation promotes the progression of Crohn's disease (CD). A more recent study has suggested that ruscogenins (RUS) can exert anti-inflammatory effects through activation of the Nrf2/NQO1 pathway. The current study is aimed at determining the functionalization of RUS on CD-like colitis. Wild-type (WT) mice induced with trinitrobenzene sulfonic acid (TNBS) exhibit a significant inflammation in their colon and are hence widely used for CD models. In the current study, the mice were treated with the Nrf-2 antagonist (ML385) or ruscogenin (RUS) whereas normal WT mice were kept as the negative control. Comparative analysis was then performed on the inflammation and barrier function of the colons. *In vitro* analysis of mouse colonic organoid systems revealed the influence of RUS on LPS-induced apoptosis, cytokine, and chemokine expressions in the intestinal epithelium. It was found that RUS ameliorates murine colitis through activation of the Nrf2/NQO1 pathway which was presented as a decrease in inflammation score and downregulated levels of cytokine and chemokine synthesis, as well as increased intestinal permeability. Further, it was noted that RUS alleviated LPS-induced apoptosis in the intestinal epithelium cells through upregulation of the Nrf2/NQO1 signaling pathway in the mouse colonic organoids. In addition, ruscogenin (RUS) attenuated the levels of Bax and C-caspase-3 through activation of the Nrf2/HO1 signaling pathway both *in vivo* and *in vitro*. Therefore, it was evident that RUS can be applied as a potential alternative therapeutic agent in CD based on its protective effects on the barrier function and anti-inflammatory activity.

## 1. Introduction

Crohn's disease (CD) is an inflammatory bowel disease whose etiology is unknown and with lethal and lifelong recurrence tendency in patients [1]. As industrialization advances, the incidence of CD is steadily increasing in the recent years [2]. Although pharmacotherapy is the mainstay for management of CD, previous studies have also shown that 50% of patients with CD still need surgical intervention for resection of diseased intestinal segments [3]. Lack of efficacious pharmacotherapy and potential drug toxicity have hindered the improvement of treatment outcomes of CD [4]. Therefore, there is an immediate demand for safe and effective therapies for clinical treatment of CD.

The dysfunction of mucosa barriers caused by abnormal immune response and infection plays a crucial role in the pathogenesis of CD whereas alteration of intestinal permeability is a major manifestation of barrier dysfunction [5, 6]. There is external material exchange during the process of intestinal absorption, and the intestinal epithelial barrier plays a critical role in blocking the entry of gut microbiota or harmful metabolites into the gut [7]. The intestinal epithelial cell presents a dynamic balance between injury and repair under pathophysiological conditions [8]. A series of physiological factors may contribute to induce intestinal hyperpermeability, and abnormal apoptosis of intestinal epithelial cells can lead to damage of the intestinal barrier as well as directly increase permeability [9]. The aberrant apoptosis of intestinal epithelial cells mainly results from aberrant activation of apoptotic pathways in response to inflammatory stimuli [10]. Therefore, upregulation of the antiapoptotic protein (B-cell lymphoma-2, Bcl-2) or downregulation of the proapoptotic protein (Bcl-2-associated X protein, Bax) may antagonize inflammation-induced apoptosis of intestinal epithelial cells and protect intestinal barrier function and this in turn ameliorates CD-like intestinal inflammation [11, 12].

The nuclear factor E2-related factor 2 (Nrf2) is a transcription factor which can coordinate the protective effects of oxidative stress in various cells, and its activity has important implications on human health [13, 14]. It has been reported that silencing Nrf2 significantly blocks the effect of drugs antagonizing apoptosis, suggesting that Nrf2 signaling is important in suppressing apoptosis [15]. Further, it has been reported that activation of the Nrf2 pathway in cells can upregulate the expression of the antiapoptotic protein Bcl-2, leading to downregulation of the expression of Bax, which in turn inhibits cell apoptosis [16]. In addition, the Nrf2 pathway is involved in intestinal ischemia-reperfusion injury and activation of this pathway can effectively alleviate intestinal mucosal injury [17]. Therefore, enhanced activation of Nrf2 can antagonize the excessive apoptosis of intestinal epithelial cells and thus protect the integrity of intestinal epithelial barrier.

Ruscogenin (RUS) is a major steroidal sapogenin found in the Chinese herb Radix Ophiopogon japonicus which is used for the treatment of acute and chronic inflammation and cardiovascular disease [18, 19]. Ruscogenin (RUS) exhibits several pharmacological properties, including improving prostate dysfunction, inhibition of microbial activity, anti-inflammation, and antitumor [20]. Ruscogenin (RUS) has a protective effect on lung epithelial injury induced by lipopolysaccharide (LPS) and significantly prevent lung endothelial cells from undergoing apoptosis [21]. Representative studies have shown that RUS as a compound of Ophiopogonis Radix can activate the Nrf2 signaling to reduce the oxidation reaction and inhibit the differentiation of fibroblasts into myofibroblasts in the process of pulmonary fibrosis in rats [22, 23]. These studies hence suggest that RUS may be a natural agonist of Nrf-2 with a function to activate the Nrf-2/NQO1 pathway in pathological process.

Although more attention has been paid by researchers on the treatment of CD by regulating Nrf2, there is still little that is known about it. Furthermore, although ruscogenin as a naturally nontoxic class of bioextraction drugs has the potential to treat CD, there is no relevant research on the relationship between CD and RUS. Therefore, it is important to explore the therapeutic effect of RUS on CD. The current study enriches the choice of treatment for CD and also promotes the understanding of the role of Nrf2 in improving the intestinal barrier function.

## 2. Materials and Methods

### 2.1. Animals

C57BL/6J mice were purchased from Jackson Lab and housed in a normal SPF environment (10 weeks old, male). The mice were randomly divided into five groups: WT group, NBS group, TNBS plus RUS group (0.5 mg/mouse), TNBS plus RUS group (1 mg/mouse), and TNBS plus RUS group (2 mg/mouse). The mice that were raised normally served as controls; for the TNBS groups, the mice were induced by TNBS and lasted for 10 days; for the TNBS plus RUS groups, mice were induced by TNBS and concurrent administration of RUS (orally once per day for 10 days). After day 10, mice were euthanized to measure the colon length and tissues from mice were collected to prepare for subsequent analyses.

### 2.2. Colitis Induced by TNBS

In the present study, the mice were intraperitoneally injected with pentobarbital (80 mg/kg). Mice were placed head first in supine position for TNBS enema slowly through a thin catheter into the colon (2.5 mg/0.1 mL 50% ethanol/0.02 kg). After extubation, mice were kept in the original position for 3 min to avoid leakage of the intracolonic instillation and were then placed on a warm bed until consciousness was regained. The induction lasted for 10 days, and enemas were performed once daily.

### 2.3. DAI Score

The mice were observed and DAI scored every other day until the end of drug administration [24]. The scoring rules were as follows: scores were calculated on a 6-point scale (0-5). An increase of 1 point is achieved according to each of the following characteristics: the hair appeared wrinkled and yellow, bloody feces, rectal prolapse less than 1 mm, and thin feces; an additional 1 point was added when diarrhea or severe rectal prolapse (greater than 1 mm) was present.

### 2.4. HE and Inflammation Score

Colon tissues were embedded in paraffin to make sections and stained with hematoxylin and eosin (H&E) to analyze morphological changes [25]. The intestinal inflammation score was performed, the score is 5 grades (0-4 points): no sign of inflammation (score of 0); a small number of cells infiltrate into the mucosal layer (score of 1); the infiltration of mononuclear cells leads to the separation of crypt and mild hyperplasia of mucosa (score of 2); a large number of inflammatory cell infiltration, mucous membrane destruction, goblet cell loss, and severe mucosal hyperplasia (score of 3); all of the above features are complicated by crypt abscesses or ulcers, and all samples were analyzed by 2 independent pathologists.

### 2.5. Imaging of Mice

Mice were weighed and anesthetized for intraperitoneal injection of 100 *μ*L L-012 (20 mmol/L, Cat #: 120-04891, Wako) [26]. After injection, the abdomen was kneaded gently for intraperitoneal diffusion of drugs. Acquisition parameters were automatically adjusted after 1 min using autoexposure option (Bruker, FX-PRO, USA); the degree of inflammation in the intestine was judged by signal intensity evaluation of bioluminescent images.

### 2.6. ELISA

The protein existing in the lysates of proximal colon tissues were extracted from the frozen samples, and the protein levels of TNF-*α* and IFN-*γ* were detected using the ELISA kits (R&D Systems, Emeryville, Cat #: MTA00B and PMIF00).

### 2.7. FITC

Mice were fasted for 4 hours before administration, fluorescein isothiocyanate (FITC)-dextran was administered through gavage (4 kDa; 600 mg/kg; Sigma, Cat #: FD40S) [27]. Mice were sacrificed after 4 hours, and blood was collected by cardiac puncture. Serum was centrifuged, and FITC levels in serum were measured by fluorescence intensity to analyze the intestinal permeability of mice.

### 2.8. 16sDNA

DNA was isolated from plasma samples using the DNeasy kit (Qiagen, Cat #: 69504); amplifications were performed using PCR using the designed primers (Panbakt 923f1: AACTCAAAGGAATTGACGGGG, Panbakt 923f2: AACTCAAATGAATT GACGGGG, and Panbakt 1124r: GCTCGTTGCGGGACT). Bacterial 16S rDNA abundance in plasma samples was analyzed using RT qPCR assay (ABI prism 7900 sequence detector, USA, Applied Biosystems) according to a previously determined standard curve.

### 2.9. Transepithelial Electric Resistance (TEER)

Fresh colonic tissues from mice were collected, and the luminal contents were gently rinsed away using PBS before placing the intestinal segments in Krebs buffer (37°C, pH: 7.33–7.37). The intestinal tube was cut along the mesentery axis and into 2.8 mm × 11 mm; the appropriate size of the intestine was put into the rectangular slider; and finally, the slider was put into the Ussing chamber system (Chamber Systems CSYS-4HA; Warner Instruments, USA). Krebs buffer was added to both chambers of the system with 10 mM glucose added to the serum side as an energy source, and 10 mm mannitol was then added to the luminal side to maintain osmolarity balance. Voltages and currents were adjusted by the system and analysis software. The tissue was allowed to equilibrate for 15 min before the readings were taken, after which the transepithelial electric resistance (TEER) was then measured.

### 2.10. Bacterial Culture

Mesenteric lymph nodes (MLN) and liver tissue samples were aseptically isolated, and two samples per tissue were extracted for bacterial culture [28]. The specific method was as follows: the collected tissue samples were weighed and mixed into 0.1 g of tissue with 0.9 mL of sterile normal saline, thoroughly ground to homogeneity. The tissue homogenates (100 *μ*L) were seeded on MacConkey agar plates (Sigma) for 24 hours in a 37°C incubator. Bacteria on plates were then counted as colony-forming units per gram of tissue, and the culture results were considered positive when there is more than 10^2^ colony units/g. The changes in intestinal permeability of mice were compared with the differences in the positive rates of each group.

### 2.11. Organoid Culture

Colonic crypts from WT mice were extracted, and the organoids were cultured with IntestiCult™ organoid growth medium (STEMCELL, Cat #: 06005), randomly divided into four groups (control, RUS, ML385 and LPS) by normal culture until day 4. The organoids in the LPS group were induced with LPS (50 *μ*g/mL, Sigma, Cat #: L4130) for 24 hours; the organoids in the RUS group were then cocultured with RUS (5 *μ*m, 10 *μ*m, and 20 *μ*m, Sigma, Cat #: SMB00295) for 24 hours at the same time as LPS induction; organoids in the ML385 group were cocultured with RUS (10 *μ*m) and ML385 (5 *μ*m, TargetMol, Cat #: T4360) for 24 hours at the same time as LPS induction. Finally, the organoids in the control group were cultured as normal.

### 2.12. Quantitative Assessment of MTT in Organoid

The MTT was executed as previously reported to measure the cell death levels in the organoids [29]. The organoids were stained with MTT, and Matrigel was dissolved in 2% SDS. The crystal of tetrazolium salt was dissolved in DMSO, and the absorbance was measured at 562 nm. The organoids from the RUS, LPS, and ML385 groups were compared with those from the control group, and cell death was measured through MTT reduction.

### 2.13. Immunofluorescence

Antibodies against Villin (1 : 100; Abcam, Cat #: ab97512 and) and active Caspase-3 (1 : 100; R&D Systems, Cat #: AF835) were used to perform immunofluorescence (IF) for observing the apoptotic epithelial cells in the mouse colon organoids.

### 2.14. Western Blotting

Western blotting (WB) was executed as previously reported to measure the protein levels in the tissues and cells [30]. Primary antibodies against Bax, Bcl-2, cleaved caspase-3, caspase-3, Nrf2, HO1, NQO1, NLRP3, caspase-1, caspase-11, GSDMD, G-CSF, RANTES, MCP1, and *β*-actin were used in the present study (Abcam; Cat #: ab32503, ab182858, ab214430, ab184787, ab92946, ab13243, ab34173, ab214185, ab138483, ab180673, ab219800, ab181053, ab189841, ab242013, and ab81283) at dilutions of 1 : 1000.

### 2.15. RT-qPCR

RT-qPCR was also executed as previously reported to measure mRNA levels in the tissues [31] of TNF-*α*, IFN-*γ*, and *β*-actin. The sequence information for gene-specific primers of mouse is exhibited in Table 1.

### 2.16. Statistical Analysis

Data were statistically analyzed using SPSS 17.0 (SPSS Inc., Chicago, IL). Independent samples *t*-test was used to compare data between two groups, and the chi-squared test was used to analyze categorical data. A *P* value of less than 0.05 was considered statistically significant.

## 3. Results

### 3.1. RUS Activated the Nrf2/NQO1 Pathway In Vitro and *In Vivo*

**Figure 1:**
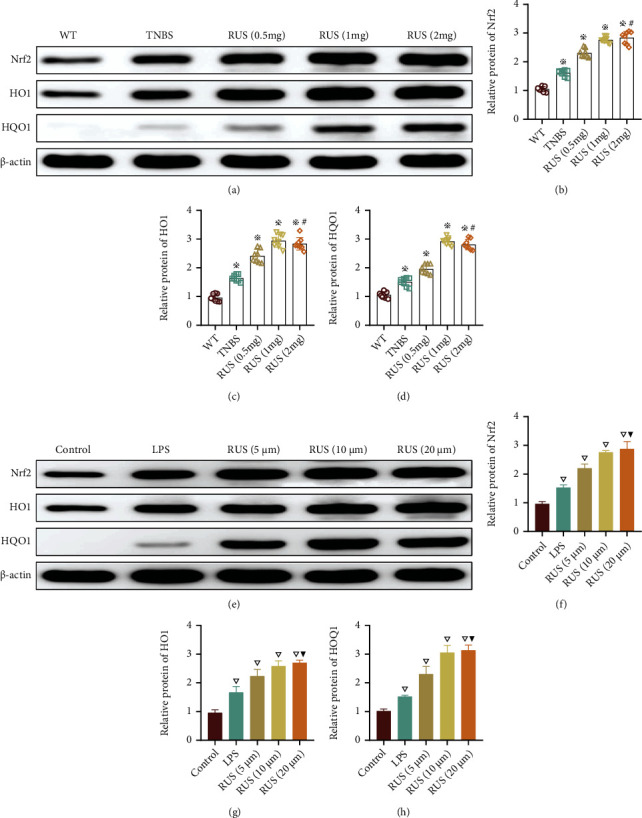
RUS activated the Nrf2/NQO1 pathway in vitro and in vivo. (a–d) Expression levels of proteins associated with Nrf2/NQO1 signaling pathways in colon mucosal samples as determined by WB. The mice in the RUS group were coadministered with TNBS and RUS. (e–h) Expression levels of proteins associated with the Nrf2/NQO1 signaling pathway in organoids from LPS-treated mice as determined by WB. Organoids in the RUS groups were simultaneously cocultured with RUS and LPS for 24 hours. The experiments were repeated 3 times (*n* = 8), and the most typical result is shown. Data are presented as the means ± SD (^※^*P* < 0.05, compared with the WT group; ^#^*P* > 0.05, compared with the RUS (1 mg) group; ^▽^*P* < 0.05, compared with the control group; ^▼^*P* > 0.05, compared with the RUS (10 *μ*m) group).

The effect of RUS on the Nrf2/NQO1 pathway was explored using a TNBS model and mouse colon organoids. Results showed that TNBS treatment elicited compensatory protection *in vivo* by activating the Nrf2/NQO1 signaling pathway. Interestingly, RUS treatment increased expression of proteins associated with the Nrf2/NQO1 pathway. The activation of the Nrf2/NQO1 pathway was higher when the concentration of RUS was high. However, the increase in Nrf2 and NQO1 expression reached a plateau at a dose of 2 mg (Figures 1(a)–1(d)). In line with this, it was found that treatment with LPS (24 hours, 50 *μ*g/mL) induced inflammatory injury in mouse colon organoids by activating the Nrf2/NQO1 pathway. The expression of Nrf2 and NQO1 proteins was higher in mice treated with RUS than in those treated with LPS. The increase in Nrf2 and NQO1 expression reached a plateau at a dose of 20 *μ*m/mL RUS (Figures 1(e)–1(h)).

### 3.2. RUS Ameliorated TNBS-Induced CD-Like Colitis

**Figure 2:**
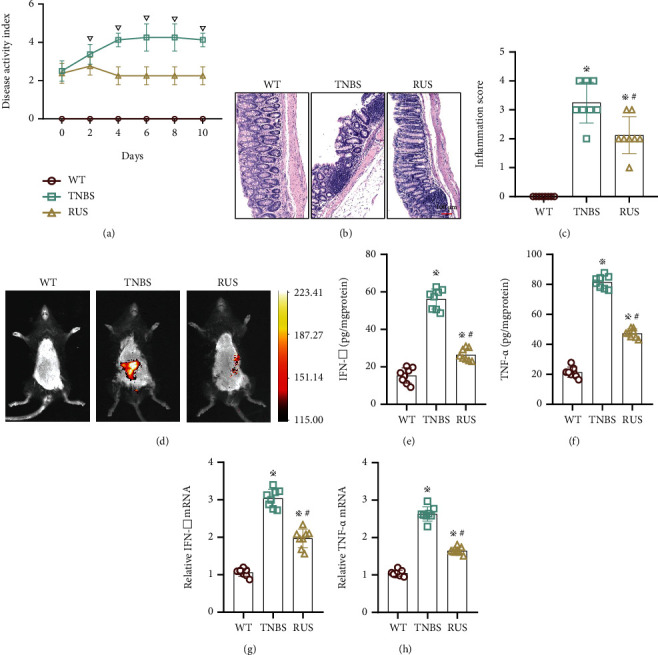
RUS ameliorated TNBS-induced CD-like colitis. (a) The DAI scores of mice in the WT group, TNBS group, and RUS (1 mg) group; the DAI scores were determined using a 6-point (0-5) scale; each mouse was evaluated daily. (b) Hematoxylin and eosin (H&E) staining of colonic tissues in each group after 10 days of treatment. Inflammation areas are indicated by arrows. Magnified images are displayed in the lower left corner of the figure. (c) The inflammatory scores of intestinal tissues in each group after treatment. (d) The degree of inflammation in the mouse intestines as revealed by L-012 autoluminescent dye. (e, f) The protein levels of TNF-*α* and IFN-*γ* in colon tissues from each group. (g, h) The mRNA levels of cytokines in colon tissues from in each group. WT mice were divided into 3 groups: WT group, TNBS group, and RUS group. The experiments were repeated 3 times (*n* = 8), and the most representative result is shown. Data are presented as the means ± SD (^※^*P* < 0.05, compared with the WT group; ^#^*P* < 0.05, compared with the TNBS group; ^▽^*P* < 0.05, compared with the RUS group).

To verify the *in vivo* therapeutic effects of RUS, experiments were performed on a TNBS model. Analysis of the DAI scores showed that TNBS treatment markedly caused higher injury and the damage was gradually aggravated over time. Administration of RUS (1 mg/day) *in vivo* alleviated the TNBS-induced damage and improved the survival of mice (Figure 2(a)). The results of HE staining and inflammation scoring revealed that *in vivo* administration of RUS reduced inflammation in colon tissues; however, the degree of inflammation in the RUS group was higher than that in WT mice (Figures 2(b) and 2(c)). Mouse live imaging (L-012) also proved that although there was still some degree of inflammation in the colon of mice in the RUS group relative to WT mice, the extent of inflammation in the RUS group was lower than that in the TNBS group (Figure 2(d)). Results of the ELISA test revealed that colon tissues of TNBS-treated mice had higher levels of inflammatory factors (TNF-*α* and IFN-*γ*), which were reduced following treatment with RUS. Similar observations were made in RT-qPCR tests (Figures 2(e)–2(h)).

### 3.3. RUS Ameliorated TNBS-Induced Intestinal Barrier Dysfunction

**Figure 3:**
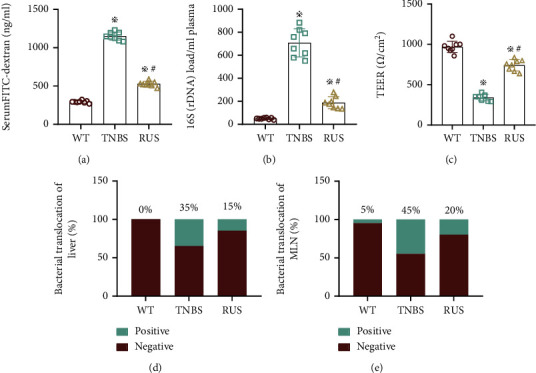
RUS ameliorated TNBS-induced intestinal barrier dysfunction. (a) The serum levels of FITC in the WT group, TNBS group, and RUS group. (b) The abundance of serum flora in the WT group, TNBS group, and RUS group. (c) The TEER values of colon tissues in the WT group, TNBS group, and RUS group. (d, e) The rate of bacterial translocation in the MLN and liver as determined in cultured bacteria. These experiments were repeated 3 times (*n* = 8), and the most representative result is shown. The data are presented as the means ± SD (^※^*P* < 0.05, compared with the WT group; ^#^*P* < 0.05, compared with the TNBS group).

To verify the protective effect of RUS on intestinal function, we further examined the intestinal barrier function in mice. FITC (4 kDa) was administered by gavage, and the concentration of FITC in serum was monitored after 4 hours. Results showed that the serum concentration of FITC was higher in TNBS-treated mice than in WT mice. In comparison, FITC serum concentration of the RUS group was lower than that of mice in the TNBS group but higher than that of WT mice (Figure 3(a)). The results of 16sDNA analysis showed that the abundance of serum flora in the TNBS group was higher than that in WT mice; mice in the RUS group had lower abundance of serum flora than mice in the TNBS group, but higher than WT mice (Figure 3(b)). Data from the TEER test showed that electrical resistance was attenuated in TNBS-treated mice relative to untreated mice, which was restored by RUS treatment (Figure 3(c)). Bacterial culture of liver and MLN samples showed that mice in the TNBS group had the highest percentage of bacterium positivity; the percentage of bacterium positivity in the RUS group was lower than that in the TNBS group, but still higher than WT mice (Figures 3(d) and 3(e)).

### 3.4. RUS Inhibits Intestinal Epithelial Cell Apoptosis but Not Pyroptosis

**Figure 4:**
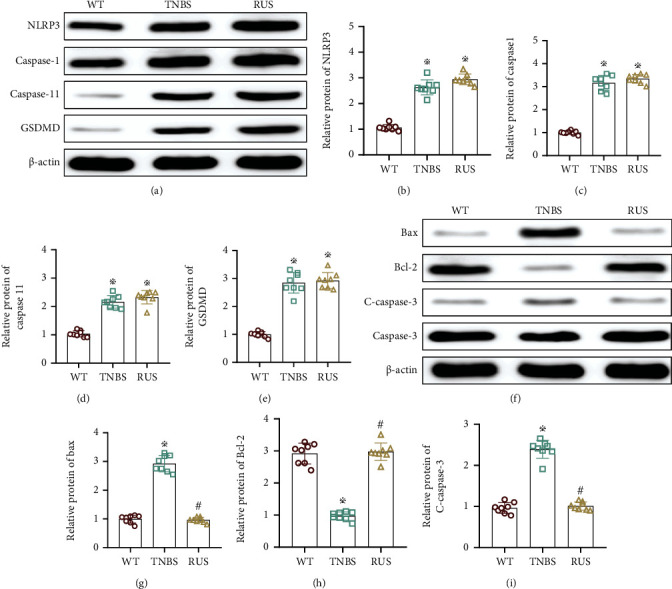
RUS inhibits intestinal epithelial cell apoptosis but not pyroptosis. (a–e) The expression of NLRP3, caspase1, caspase11, and GSDMD in colon tissues as determined by WB. (f–i) Protein levels of Bcl-2, Bax, and C-caspase-3 in colon tissues. The experiments were repeated 3 times (*n* = 8), and the most typical result is shown. Data are presented as the means ± SD (^※^*P* < 0.05, compared with the WT group; ^#^*P* > 0.05, compared with the WT group).

To determine the effects of RUS on epithelial cells, pyroptosis and apoptosis signaling pathways were investigated. Data showed that TNBS treatment activated the pyroptosis pathway in mouse epithelial cells and upregulated expression of protein markers (NLRP3, caspase1, caspase11, and GSDMD). However, the expression of these proteins was not downregulated as expected by the RUS intervention compared with the TNBS group (Figures 4(a)–4(e)). It was exciting that the apoptosis pathway in mouse epithelial cells was activated obviously after TNBS induction, and the level of related apoptotic protein (Bax and C-caspase-3) had been upregulated in a synchronous manner; the expression of antiapoptotic protein Bcl-2 was downregulated; the expression of Bax and c-caspase-3 was downregulated, and the expression of Bcl-2 was upregulated by the intervention of RUS (Figures 4(f)–4(i)). It indicated that RUS could protect the function of the intestinal barrier by inhibiting the apoptosis of epithelial cells.

### 3.5. RUS Ameliorated Inflammatory Responses in Intestinal Epithelial Cells

**Figure 5:**
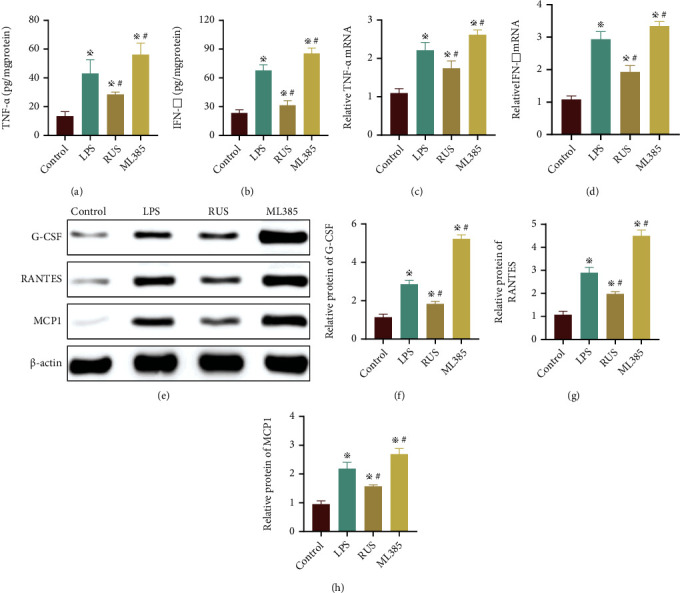
RUS ameliorated inflammatory responses in intestinal epithelial cells. (a, b) The protein levels of TNF-*α* and IFN-*γ* in organoids collected from the control group, LPS group, RUS group, and ML385 group (with RUS). (c, d) The mRNA levels of cytokines in organoids collected from the control group, LPS group, RUS group, and ML385 group. (e–h) The protein levels of G-CSF, RANTES, and MCP1 in organoids from the control group, LPS group, RUS group, and ML385 group as determined by WB. The experiments were repeated 3 times (*n* = 8), and the most typical result is shown. Data are presented as the means ± SD (^※^*P* < 0.05, compared with the control group; ^#^*P* < 0.05, compared with the LPS group).

To explore whether RUS ameliorated the inflammatory response of epithelial cells via the Nrf2/NQO1 pathway, mouse colon organoids were used to mimic the *in vivo* environment. In this experiment, LPS (24 hours, 50 *μ*g/mL) was administered to induce inflammatory injury in organoids but we found that the expressions of inflammatory factors (TNF-*α* and IFN-*γ*) were all enhanced. The expression of these inflammatory factors was downregulated by RUS (10 *μ*mol) treatment. Of note, the protective effect of RUS was antagonized by ML385 (5 *μ*mol) which inhibits Nrf2, as validated by RT qPCR (Figures 5(a)–5(d)). The results of western blotting showed that the expression of macrophage migration-related factors (G-CSF, RANTES, MCP1) in organoids was all upregulated by LPS induction. The expression of macrophage migration-related factors was downregulated by RUS intervention, which was antagonized by simultaneous administration of ML385 (Figures 5(e)–5(h)).

### 3.6. RUS Improved Intestinal Epithelial Barrier Function by Inhibiting Epithelial Cell Apoptosis

**Figure 6:**
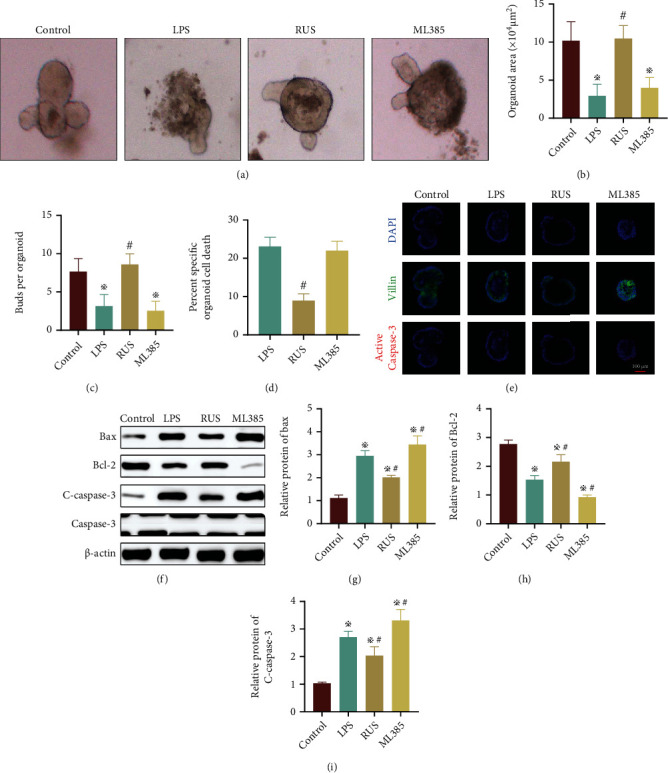
RUS improved intestinal epithelial barrier function by inhibiting epithelial cell apoptosis. (a) Growth of organoids in the control group, LPS group, RUS group, and ML385 group (with RUS) as observed by a light microscope. (b, c) The size and buds of organoids in each group. (d) MTT assay showing the activity of organoids. (e) Immunofluorescence analysis showing the distribution of Villin (green) and active Caspase-3 (red) in organoids. DAPI (blue) was used to stain the nuclei. (f–i) The protein levels of Bcl-2, Bax, and C-caspase-3 in organoids as determined by WB.^✲^*P* < 0.05, compared with the control group. The experiments were repeated 3 times, and the most typical result is shown. Data are presented as the means ± SD (^※^*P* < 0.05, compared with the control group; ^#^*P* < 0.05, compared with the LPS group).

To further define whether RUS improved apoptosis of epithelial cells via the Nrf2/NQO1 pathway, we examined the proliferation and apoptosis level of organoids. Analysis of the organoid area size and budding revealed that organoids in the LPS group were smaller in size and formed few buds; and this was improved by RUS treatment. However, ML385 treatment abolished the protective effect of RUS, and the size and budding of organoids were similar to those of the LPS group (Figures 6(a)–6(c)). The MTT assay revealed that organoids treated with LPS had more dead cells compared to normal cultures, whereas those treated with RUS had a lower number of dead cells. ML385 treatment resulted in fewer numbers of dead cells compared to LPS treatment (Figure 6(d)). Examination of localization by immunofluorescence (Villin and C-caspase-3) showed that cells treated with LPS had marked apoptosis levels, which were ameliorated by RUS treatment. In addition, cells treated with ML385 showed fewer numbers of apoptotic cells compared to those treated with LPS (Figure 6(e)). Moreover, treatment with LPS upregulated the expression levels of apoptosis-related proteins (Bax and c-caspase-3) but decreased the expression of Bcl-2in the organoids. These changes in the expression of apoptosis-related proteins were reversed by RUS treatment, but this effect was antagonized by concomitant administration of ML385 (Figures 6(f)–6(i)).

## 4. Discussion

To the best of our knowledge, this study is the first to show that RUS activates the Nrf2/NQO1 pathway to ameliorate CD-like colitis in TNBS-treated mice. The main findings are as follows: (1) RUS ameliorates CD-like colitis by upregulating the expression of proteins associated with the Nrf2/NQO1 pathway; (2) RUS suppresses inflammatory responses in intestinal epithelial cells; and (3) RUS inhibits apoptosis of intestinal epithelial cells to protect the integrity of the intestinal barrier.

Therefore, this study shows that RUS confers protection against TNBS-induced enteritis. Intestinal barrier dysfunction is associated with high expression of cytokines. Here, we found that RUS treatment decreased inflammatory scores, DAI, and the levels of cytokine in the colon of mice. Therefore, the indicators associated with changes in the intestinal barrier function were analyzed. It was found that RUS reversed the increase in bacterial abundance in serum induced by TNBS and improved the permeability of the intestinal barrier. The abundance of bacteria in serum influences the intestinal barrier integrity, and the abundance of 16S flora partially reflects the degree of intestinal inflammation [32]. Our data suggested that RUS prevented TNBS-induced damage by improving the epithelial barrier to some extent. Disturbance of the intestinal epithelial barrier function increases intestinal permeability in CD [33]. Herein, we found that the level of FITC in serum and the likelihood of bacterial translocation were significantly reduced in the RUS group. These data indicated that RUS conferred protection against CD.

Rus can activate the Nrf2 pathway, which may have therapeutic significance in CD-like enteritis [22, 34]. Numerous studies have demonstrated that activation of the Nrf2 pathway inhibits the Bax/Caspase-3 signaling pathway [35]. Aberrant apoptosis of intestinal epithelial cells contributes to functional disruption of the intestinal barrier [36]. Our results showed that administration of RUS enhanced the Nrf2/NQO1 signaling pathway and inhibited the activation of the classical apoptotic pathway. Interestingly, several components of the Nrf2/NQO1 signaling pathway prevent apoptosis of intestinal epithelial cells [37]. The activation of Nrf2 in this study promoted the functional recovery of the intestinal barrier by increasing the expression of Bcl-2 and reducing apoptosis of epithelial cell following LPS treatment, which might explain why RUS ameliorated TNBS-induced enteritis in WT mice. The Nrf2/NQO1 signaling pathway has been shown to be critical for the functional recovery of the intestinal barrier [38]. The protective effect of RUS on the intestinal barrier might be partly due to the activation of Nrf2/NQO1 signaling, which inhibits the Bax/c-caspase-3 apoptotic pathway. However, it is likely that this protective effect of RUS may also be related to its antioxidant and anti-inflammatory properties, rather than direct regulation of apoptosis.

Despite these useful findings, this study still has some limitations. For example, the results proved that LPS increased Nrf2 expression as a compensatory response. RUS further promoted the expression of Nrf2 to execute its protective effects. However, ML385 completely inhibited the expression of Nrf2, which may explain the stronger activation of the Bax/C-caspase-3 apoptotic pathway in the ML385 group. The results showed that RUS activated the Nrf2/NQO1 pathway, but the mediators involved need to be investigated. In addition, RUS promoted functional recovery of the intestinal barrier in CD by ameliorating inflammatory responses and inhibiting apoptosis in epithelial cells. However, RUS may also improve CD through other means. Attenuation of the Bax/c-caspase-3 signaling pathway may explain the positive effects of RUS on the barrier function, but whether other signaling pathways are involved needs to be determined since RUS may regulate several pathways.

In conclusion, this study demonstrates that RUS ameliorates the pathophysiological processes associated with CD-like colitis in TNBS-induced mice by suppressing cytokine expression in intestinal epithelial cells and inhibiting the levels of apoptotic proteins in epithelial cells through the Nrf2/NQO1 pathway. RUS may improve the intestinal barrier function by activating the Nrf2/NQO1 signaling pathway and increasing expression levels of the antiapoptotic protein Bcl-2 in intestinal epithelial cells. These data demonstrate that RUS confers protection against CD and is likely to be a new therapeutic agent for CD treatment.

## Figures and Tables

**Table 1:** Primer sequences (5′ to 3′).

Gene name	Forward primer	Reverse primer
*Tnf-α*	CAGGCGGTGCCTATGTCTC	CGATCACCCCGAAGTTCAGTAG
*Ifn-γ*	ACAGCAAGGCGAAAAAGGATG	TGGTGGACCACTCGGATGA
*β-Actin*	GATTACTGCTCTGGCTCCTAGC	GACTCATCGTACTCCTGCTTGC

## Data Availability

The data can be obtained from the corresponding author on request.

## Abbreviations

CD: Crohn's disease
DAI: Disease activity index
WT: Wild-type
LPS: Lipopolysaccharide
Nrf2: Nuclear factor E2-related factor 2
MTT: 3-(4,5-Dimethylthiazol-2-yl)-2,5-diphenyltetrazolium bromide
TNBS: Trinitrobenzene sulfonic acid
HO1: Heme oxygenase 1
MLN: Mesenteric lymph node
TNF-*α*: Tumor necrosis factor alpha
FITC: Fluorescein isothiocyanate
RUS: Ruscogenins
TEER: Transepithelial electric resistance.
